# Structural characterization and macromolecular structure construction of non-caking coal in Chicheng Mine

**DOI:** 10.1038/s41598-023-44045-2

**Published:** 2023-10-07

**Authors:** Jinzhang Jia, Qiang Yang, Baogang Liu, Dongming Wang

**Affiliations:** 1 College of Safety Science and Engineering, Liaoning Technical University, Fuxin, 123000 Liaoning China; 2 Key Laboratory of Mine Thermodynamic Disasters and Control of Ministry of Education (Liaoning Technical University), Huludao, 125105 Liaoning China

**Keywords:** Chemistry, Energy science and technology, Engineering

## Abstract

Given that coal is not a homogeneous substance, it is of great significance to identify and quantify the structural characteristics thereof for comprehensive analysis. In this paper, the non-caking coal in Chicheng Coal Mine was selected as the research object. The C, H, O, N, and S contents in the coal samples were obtained by proximate/ultimate analysis experiments. The structural characteristics of aromatic, aliphatic hydrocarbons, and oxygen-containing functional groups in the experimental coal samples were obtained by Fourier transform infrared spectroscopy (FT-IR) experiments. X-ray photoelectron spectroscopy (XPS) experiments were performed to obtain the occurrence state of N and S elements in coal samples. Nuclear magnetic resonance (^13^C NMR) experiments provided the carbon skeleton structural information of the coal samples. The macromolecular structure model of non-caking coal in the Chicheng Coal Mine was established on this basis. The molecular formula of non-caking coal in Chicheng Coal Mine was determined to be C_207_H_181_O_32_N_3_S.The^13^C NMR-predicted spectrum of the model showed good consistency with the ^13^C NMR-experimental spectrum obtained from the experiments, providing researchers with reference to the construction ideas and methods of molecular structure models of different coal samples.

## Introduction

The macromolecular structure of coal is complex, and significant molecular structure and morphological differences exist for coals at different metamorphic degrees^1^. As such, it is imperative to build an accurate macromolecular structure model when studying the adsorption-diffusion of coal bed methane in coal. The structure of coal is closely related to its adsorption and reactivity properties, therefore coal structure is one of the main topics in coal chemistry research. The correct understanding of the occurrence status and microcrystalline structure parameters of the main elements in coal is of practical significance for constructing macromolecular structure models, studying the inherent properties of coal, achieving the "dual carbon" goal, and achieving high-quality development of the coal industry.

Initially, Fuchs^2^ established the first macromolecular structure model of lignite by structural statistical analysis, which could quantitatively describe the aromatic and fatty clusters in coal structure. This provided the foundation for experimental research and theoretical analysis for developing the macromolecular structure model of coal. Given^3^ used infrared spectroscopy and X-ray diffraction methods to evaluate the ratio of aromatic hydrogen to aliphatic hydrogen and the composition of functional groups. The molecular structure model of bituminous coal with condensed aromatic structure mainly composed of benzene and naphthalene was constructed on this basis. However, the deficiency of this model was that the existence of S atoms and ether bonds was not considered, so it could not accurately reflect the chemical properties of low metamorphic bituminous coal.

Wiser^4^ constructed a coal macromolecular structure model with an aromatic structure (aromatic rings were cross linked by weak bonds such as ether bond, thioaldehyde bond, and short burning bond) and added the S element to the model. The Wiser model is considered a modern coal model that can comprehensively reflect the fundamental structure of coal molecules. Honda model^5^ was based on the aromatic structure of phenanthrene and naphthalene. Short bonds such as –CH_2_ and –CH were used as cross linking structures to construct the Honda macromolecular structure model, which considers the existing forms of small molecular compounds and macromolecular compounds in coal molecular structure. Shinn^6^ constructed the Shinn bituminous coal macromolecular structure model based on the functional groups and bridge bond types on the coal's surface. The occurrence forms of sulfur, ammonia, and other hetero atoms in the structure were considered and recognized by most researchers.

In recent years, with the rapid progress of physical and chemical characterization testing technology, molecular simulation technology, and computer-aided technology, the coal molecular structure model development has been further promoted. Zhang et al.^7^ studied the molecular structure of coal using limit analysis, ^13^C nuclear magnetic resonance spectroscopy, Fourier-transform infrared spectroscopy, and gas-chromatograph/mass-spectrometry analyses and established the three-dimensional substructure and physicochemical structure model of Tunlan coal. After further analysis, it was found that the nanoporous structure’s angular and torsional energy played a more important role in maintaining a stable three-dimensional structure. However, electrostatic energy in non-bond energy can form nano-pore space in coal. With the dissolution of low molecular weight compounds, the pore volume of micropores gradually increases, while the corresponding specific surface area first decreases and then increases, indicating that pore expansion and enlargement are the main effects in the early and late dissolution, respectively.

Xiang et al.^8^ established the macromolecular structure model of Yanzhou coal by analyzing the ^13^C CP/MAS NMR data of Yanzhou coal. In this model, benzene was the main aromatic compound, and the aliphatic structure mainly existed in the form of aliphatic side chains, cycloalkanes, and hydrogenated aromatic rings. Zhang et al.^9^ studied the molecular structure of Yangchangwan sub-bituminous coal by using approximate analysis, chemical composition analysis, FT-IR, C^13^ NMR, and XPS analyses and obtained key information about elements and chemical bonds in the coal structure. The aromatic hydrocarbon content of the studied coal was 71.33%, and the ratio of bridge carbon to surrounding carbon was 0.32, indicating that the proportion of naphthalene in the coal structure is higher than that of anthracene and benzene. Oxygen mainly existed as carbonyl, ether, and carboxyl functional groups, nitrogen occurred in pyridine and pyrrole, and methyl mainly existed in cyclic and aliphatic hydrocarbons. Finally, the molecular formula of Yangchangwan sub-bituminous coal was determined as C_323_H_232_O_42_N_4_S.

Zhang et al.^10^ used elemental analysis, high-resolution transmission electron microscopy, laser desorption time-of-flight mass spectrometry, C^13^ nuclear magnetic resonance spectroscopy, and X-ray photoelectron spectroscopy to construct and study the molecular model of Xishan bituminous coal. The model consisted of 62 independent molecules, the molecular formula of which was C_7972_H_4882_O_115_N_50_S_30_, which is reasonable and consistent with the structure and molecular properties determined by experiments. This is the first bituminous coal model with different molecular weights and structures in China, which is helpful to understand further the structural relationship of coal at the molecular level.

Wang et al.^11^ obtained the microstructure information of metallic coal char using high-resolution transmission electron microscope experiments and proposed a method for quickly coupling stacking and pore size distribution of coal char macromolecules, which has strong controllability and is convenient for coupling complex coal macromolecules. Gao et al.^12^ studied the pyrolysis mechanism of coal based on molecular dynamics simulation. They prepared an artificially constructed large coal model with various chemical structures from the characterization data obtained by approximate and final analyses, C^13^ NMR, and solvent extraction experiments. The model contained 23,898 atoms and consisted of 20 macromolecules with different structures and 29 small compounds. The model had a high applicability to explore the mechanism of coal pyrolysis in ReaxFF-MD simulation.

This article aims to use proximate/ultimate analysis, FTIR, XPS, ^13^C-NMR and other testing methods to study the structural parameters of Chicheng non caking coal, obtain the key information parameters of its functional groups, the occurrence states of nitrogen and sulfur elements, obtain the occurrence states of functional groups in the coal, and obtain the occurrence forms of nitrogen and sulfur organic states. Comprehensively and deeply understand the structural characteristics of Chicheng non caking coal, construct a coal macromolecular structure model, and provide a model basis for subsequent research on the spontaneous combustion tendency and adsorption capacity of Chicheng non caking coal.

## Experimental

Studying coal’s physicochemical structure can provide a full understanding of the gas adsorption-diffusion performance of coal^13^. The organic matter in coal samples was characterized and tested by proximate/ultimate analysis, FT-IR, XPS, and solid ^13^C NMR. Parameters such as coal’s aromatic ring structure, oxygen-containing functional groups, fat structure, and element occurrence state were obtained. A coal macromolecular structure model was constructed based on these data.

### Experimental equipment

**Figure 1:**
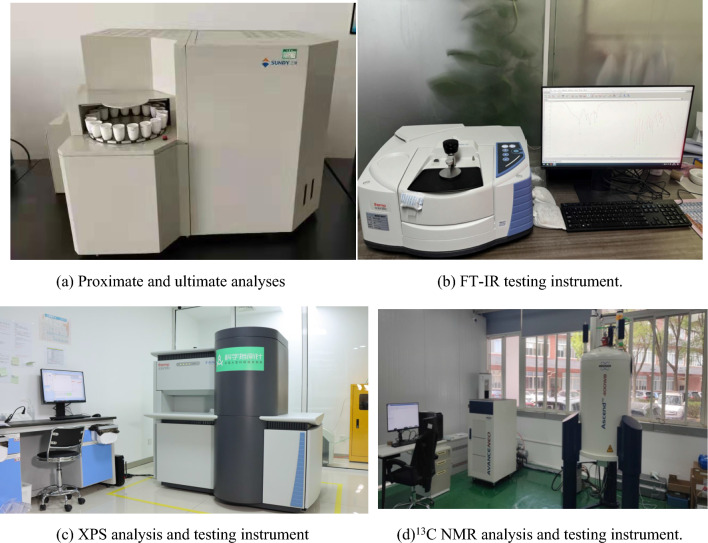
Different analytical instruments used in the study.

The coal sample characterization test instruments used in this paper are shown in Fig. 1.

The HXG5005 proximate analyzer is used to determine the ash, fixed carbon, moisture, and volatile content of coal. The relative content of major ultimate such as C, H, O, N, P, S, etc. in coal was tested using the ELTRACS-2000 ultimate analyzer. The Tensor27 infrared spectrometer from Bruker, Germany was used for testing. Take an appropriate amount of coal sample and mix it with potassium bromide in a ratio of 1:200, thoroughly stir and grind. Use a tablet press to press into transparent sheets of 0.2–0.5 mm. Place the pressed sheets in an oven at 110 ℃ for continuous drying for 4 h before removing them. Place the dried sheets into an infrared spectrometer for measurement. The infrared spectrum analyzer has a measurement range of 4000–400 cm^−1^, a resolution of 4.0 cm^−1^, and a scanning frequency of 32 times. Using Thermo Scientific™ Nexsa™ X-ray photoelectron spectrometer, the vacuum level in the analysis room is 8 × 10^−10^ Pa, excitation source using Al Ka rays (h*v* = 1486.6 eV), working voltage of 12.5 kV, filament current of 16 mA, and signal accumulation for 10 cycles. The full spectrum of the test energy is 100 eV, the narrow spectrum is 30 eV, the step size is 0.1 eV, the residence time is 40–50 ms, and the charge correction is performed using the C1s = 284.80 eV binding energy as the energy standard. ^13^C-NMR technology can obtain carbon atom skeleton information of coal without damaging its molecular structure. The experimental instrument adopts a 600 MHz solid-state nuclear magnetic resonance spectrometer from Bruker AVANCE III HD in Switzerland, with an H/X dual resonance solid probe, a 4 mm ZrO_2_ rotor, a rotational speed of 5 kHz, a detection resonance frequency of 100.625 MHz at ^13^C, and a sampling time of 5.12 μs. Spectral width 50 kHz, cyclic delay time 6.5 μs.

**Figure 2:**
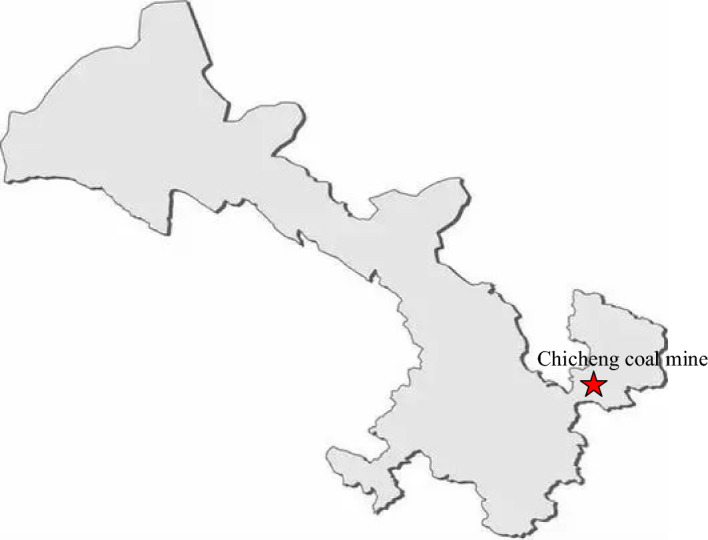
Geographical location information of Chicheng coal mine.

**Figure 3:**
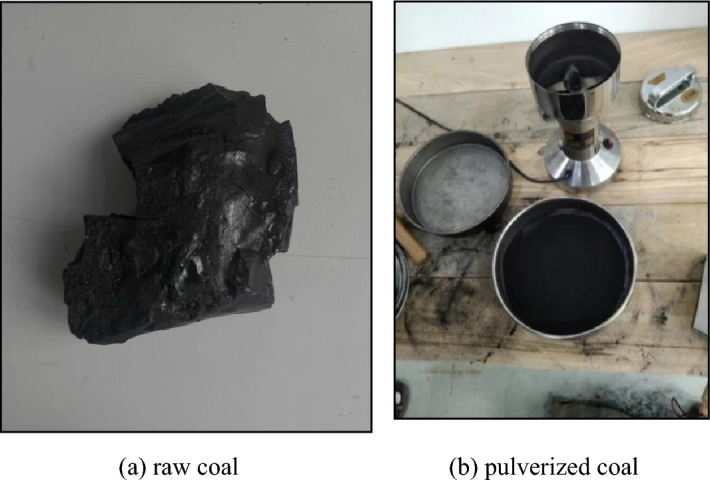
Test coal samples.

Prepare coal samples in accordance with the provisions of the "Method for preparation of coal samples" (GB474-1996). Use crushers and vibrating screens to crush, screen, and shrink anthracite, coking coal, and long flame coal, and prepare analytical samples with particle sizes below 200 mesh. The samples in this paper were selected from fresh coal samples (density 1.16 g/cm^3^, ${R}_{\mathrm{max}}^{^\circ }$ 0.665%) in the 1502-2 working face of Chicheng Coal Mine. The coal sample selected in this article is from Chicheng village, Xinyao town, Chongxin county, Pingliang, Gansu province, China. The geographical location is shown in Fig. 2. Coal samples were collected according to the Method of Coal Sampling in Coal Seam (GB/T482-2008) and prepared according to the Method of Coal Sample Preparation (GB474-1996). The coal samples were crushed, screened, and shrunk by a crusher and vibrating screen machine. The samples for analyses with particle sizes less than 200 mesh were prepared, as shown in Fig. 3. Two 20 g portions for proximate and ultimate analyses testing, 5 g triplicate for FT-IR, XPS, and solid ^13^C NMR analysis.

#### Calculation of FT-IR structural parameters of coal samples

The ordinate of the FT-IR spectrum obtained in the experiment is transmittance, which is converted into light absorption intensity by Lambert–Beer law and can be expressed as:

1 $$Y = \lg \left( {1/X} \right)$$

formula: Y is the light absorption intensity, and X is the transmittance.

Aromatic carbon rate, degree of condensation, and length of fatty chain are some basic parameters for constructing the coal macromolecular structure model. Based on the research of previous studies^13^, this paper uses peak area to quantitatively analyze the structural parameters of coal.

1. Length of the fatty chain of coal samples
  The aliphatic hydrocarbon chain length is one of the structural parameters of coal, which is expressed by the ratio of –CH_3_ to –CH_2_, as shown in Formula (2). The smaller this parameter, the longer the aliphatic chain of coal^13^. According to the calculation, the fatty chain length of the coal sample is 0.17.
  2 $$\frac{{A_{1} \left( {CH_{2} } \right)}}{{A_{1} \left( {CH_{3} } \right)}} = \frac{{A_{1} \left( {{2963}\;{\text{cm}}^{ - 1} } \right)}}{{A_{1} \left( {{2853}\;{\text{cm}}^{ - 1} } \right) + A_{1} \left( {{2918}\;{\text{cm}}^{ - 1} } \right)}}$$
  formula: A_1_ is the peak fitting area of the fourier wave number in the corresponding interval, dimensionless.
2. Aromaticity of coal sample (I)
  Aromaticity can describe the richness of aromatic to fatty functional groups, which can be expressed as^13^:
  3 $$I = \frac{{A_{1} \left( {900{ - }700\;{\text{cm}}^{ - 1} } \right)}}{{A_{1} \left( {3000{ - }2800\;{\text{cm}}^{ - 1} } \right)}}$$
  According to the above formula, the coal sample aromaticity can be 0.83.
3. Degree of condensation of aromatic rings (DOC)
  The ratio of the out-of-plane deformation vibration of the aromatic ring CH in the region with a condensation degree of 900–700 cm^−1^ to the vibration of the aromatic C=C skeleton near the region with a peak of 1600 cm^−1^ can be expressed as:
  4 $$DOC = \frac{{A_{1} \left( {900{ - }700\;{\text{cm}}^{ - 1} } \right)}}{{A_{1} \left( {1600\;{\text{cm}}^{ - 1} } \right)}}$$
  According to the above formula, the condensation degree of the coal sample's aromatic ring can be 0.18.
4. Infrared aromatic carbon rate ($f_{ar}^{C}$)
  Infrared aromatic carbon rate refers to the proportion of carbon atoms in the aromatic carbon composition of coal to the total carbon atoms^24^, and the aromatic carbon rate of coal can be calculated by formula (5)^13^.
  5 $$f_{ar}^{C} = 1 - {{{\left( {\frac{{A_{1} \left( {2800{ -} 3000} \right){\text{cm}}^{ - 1} }}{{A_{1} \left( {700 {-} 900} \right){\text{cm}}^{ - 1} + A_{1} \left( {2800 {-} 3000} \right){\text{cm}}^{ - 1} }} \times \frac{H}{C}} \right)}} {\left/ {{{\left( {\frac{{A_{1} \left( {2800 {-} 3000} \right){\text{cm}}^{ - 1} }}{{A_{1} \left( {700 {-} 900} \right){\text{cm}}^{ - 1} + A_{1} \left( {2800{ - }3000} \right){\text{cm}}^{ - 1} }} \times \frac{H}{C}} \right)} {\frac{{H_{{{\text{al}}}} }}{{C_{{{\text{al}}}} }}}}}\right.} {{\frac{{H_{{{\text{al}}}} }}{{C_{{{\text{al}}}} }}}}}$$
  formula: *H*_al_/*C*_al_ is the ratio of H and C in the aliphatic group, which is 1.8.
  According to the above formula, the infrared aromatic carbon rate of the coal sample can be 0.7764.

### Proximate/ultimate analysis

The coal sample selected in this article is high volatile A non-caking coal, obtained according to the American Society for Testing and Materials (ASTM) standard.According to the international standard for coal classification ISO11760 as shown in Table1, which is a genetic type classification, ISO11760 uses the degree of coal metamorphism (represented by vitrinite reflectance), petrographic composition (represented by vitrinite content), and coal grade (represented by dry ash yield) as the basis for coal classification. Based on proximate analysis, ultimate analysis, and vitrinite reflectance, Chicheng non caking coal in this article is a low-rank coal.In order to obtain the real industrial analysis data of the coal samples studied in this article, no deash treatment was carried out on the raw coal, and the obtained ash content data is the real raw coal. Proximate/ultimate testing and analysis is the primary method of coal structure analysis and testing^13^. In this paper, the proximate and ultimate analyses of coal samples were carried out according to the corresponding national standards (GB/T212-2008 and GB/T476-2008, respectively). The proximate and ultimate analytical results of coal samples are shown in Table 2.

**Table 1:** ISO and ASTM standard.

Project	ISO standard	ASTM standard
Collect coal samples	ISO13909-2001(E)	ASTMD2234//2234M-07
Making coal samples	ISO13909-2001(E)	ASTMD293-93(2004)
Granularity	ISO1953-1994	ASTMD346-2004
Moisture	ISO589:2008(E)	ASTMD197-1987(2007)

**Table2:** Proximate and ultimate analyses of coal.

Coal sample	Proximate analysis (%)				Ultimate analysis (wt%, daf)				
	M_ad_	A_ad_	V_ad_	FC_ad_	C	H	O^a^	N	S
	4.69	15.39	25.10	54.82	77.21	4.76	15.66	1.32	1.05

^a^Calculate the oxygen content using the subtraction method.

## Results and discussion

### Fourier transform infrared spectroscopy analysis

The type and relative content of functional groups contained in coal can be obtained by infrared spectroscopyexperiments, which have been widely used to characterize coal molecular structure. The greater the intensity and area of the infrared spectrum absorption peak of functional groups in coal, the higher the content of functional groups^14^. According to the related literature^13–23^, the FT-IR spectrum of coal is divided into four absorption bands: 3600–3000 cm^−1^ band is the hydroxyl absorption band; 3000–2800 cm^−1^ band is the aliphatic hydrocarbon absorption band; 1800–1000 cm^−1^ band is the absorption band of oxygen-containing functional groups and some aliphatic hydrocarbons; 900–700 cm^−1^ band is the aromatic hydrocarbon absorption band^24^.

#### Analysis of absorption band of aromatic structure

**Figure 4:**
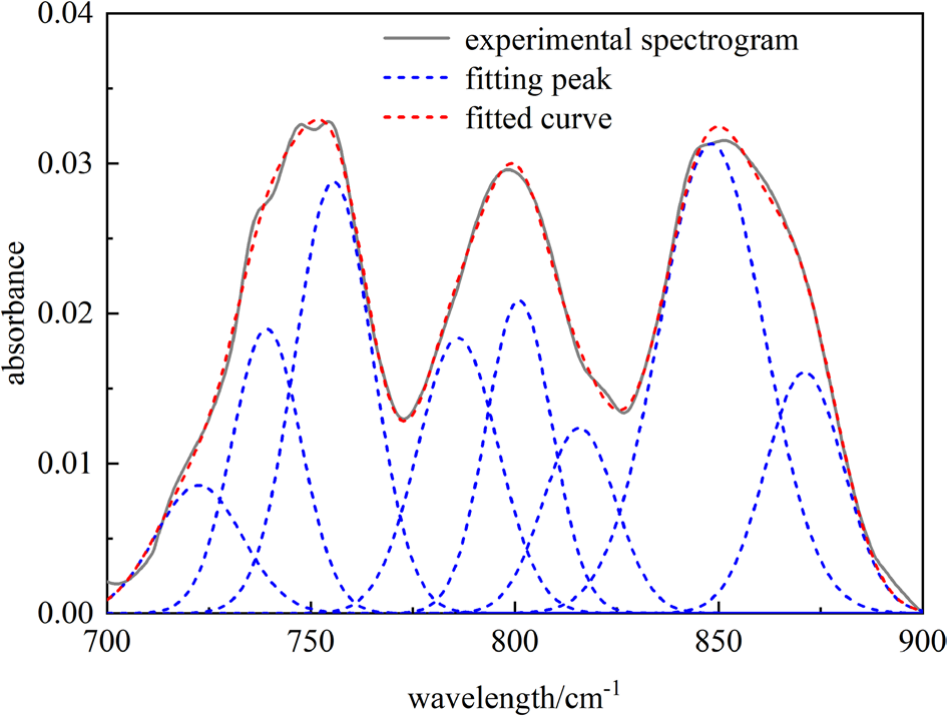
Fourier transform infrared spectroscopy fitting spectra of coal samples in the 700–900 cm^−1^ band.

Using data converted from formula (1), the ORIGIN software wasutilized to fit the spectra of coal samples in the band of 700–900 cm^−1^. After baseline correction, the second derivative of the spectra was selected to determine the approximate position and number of the initially fitted peaks^25^. Then, a Gaussian model was used to fit and optimize the peaks to obtain stable and robust information, such as the position, area,and relative content of absorption peaks, as shown in Fig. 4 and Table 3.

**Table 3:** Infrared absorption peak parameters of aromatic structure of coal.

Peak number	Peak position (cm^−1^)	Peak area	Proportion (%)	Functional group
1	722.50	0.2237	5.8191	Benzene ring disubstituted (4H)
2	739.15	0.4012	10.4361	Benzene ring disubstituted (4H)
3	755.59	0.6802	17.6917	Benzene ring disubstituted (4H)
4	786.10	0.4518	11.7514	Trisubstitution of benzene ring (3H)
5	801.06	0.4204	10.9345	Trisubstitution of benzene ring (3H)
6	815.86	0.2748	7.1489	Trisubstitution of benzene ring (3H)
7	848.25	1.0068	26.1872	Tetrasubstitution of benzene ring (2H)
8	871.07	0.3856	10.0310	Five-substituted benzene ring (1H)

The fitting results show that the main absorption peaks of aromatic structures in the coal samples are near the regions of 750 cm^−1^, 800 cm^−1^, and 870 cm^−1^, which are similar to those reported in references^26—28^. Three strong peaks appear in the experimental spectra in this region, indicating that the aromatic ring structure content in coal is relatively high. The three prominent absorption peaks correspond to disubstituted, trisubstitution, tetrasubstitution, and pentasubstituted benzene rings, and the aromatic hydrocarbon structure of coal samples is dominated by disubstituted benzene rings, accounting for about 33.95%. Tri-(tetra-) substitution of the benzene ring is the supplement, accounting for about 29.83% (26.19%); The content of pentasubstituted benzene ring is low, accounting for about 10.03%. The ratio of 2, 3, 4, and 5 substitutions of the benzene ring is about 0.34:0.3:0.26:0.1, which shows many aromatic C–H structures in coal, and the degree of aromatic ring substitution and condensation is low. Due to the low metamorphic degree of the studied coal samples, the condensation degree of the coal samples is also low, which further explains the rationality of the experimental test. Our results can quantitatively analyze the number of different substitution methods of benzene rings in the coal macromolecular structure and provide a theoretical basis for accurately constructing coal macromolecular structure.

#### Analysis of absorption bands of oxygen-containing functional groups

**Figure 5:**
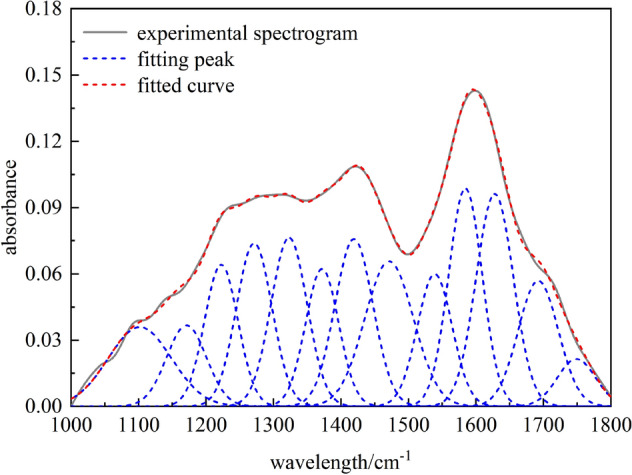
Fourier transform infrared spectroscopy fitting spectra of coal samples in 1000–1800 cm^−1^ band.

The 1800–1000 cm^−1^ band of Fourier infrared spectroscopy is an oxygen-containing functional group and some aliphatic hydrocarbon absorption bands. Among them,oxygen-containing functional groups are mainly composed of –COOH, –OH, carbonyl, and active ether bonds. Their occurrence and distribution directly affect the reaction characteristics, such as coal adsorption. The results of fitting the absorption peaks of non-caking coal in 1000–1800 cm^−1^ band region are shown in Fig. 5^18^. The functional groups of references^19,29^ and the corresponding fitting parameters are shown in Table 4.

**Table 4:** Infrared spectral absorption peak parameters of oxygen-containing functional groups in coal.

Peak number	Peak position (cm^−1^)	Peak area	Proportion (%)	Functional group
1	1098.95	4.1618	7.1325	C–O–C vibration
2	1171.08	2.7060	4.6375	C–O vibration of phenol, alcohol, ether, and ester
3	1222.58	3.9767	6.8153	C–O vibration of phenol, alcohol, ether, and ester
4	1271.39	4.8948	8.3887	C–O vibration of phenol, alcohol, ether, and ester
5	1323.10	5.1272	8.7870	C–O vibration of phenol, alcohol, ether, and ester
6	1372.10	3.8298	6.5636	CH_3_^−^ bending vibration
7	1419.26	5.3578	9.1826	Deformation vibration of CH_3_^−^ and CH_2_^−^
8	1471.40	5.9374	10.175	Deformation vibration of CH_3_^−^ and CH_2_^‒^
9	1539.0	3.8720	6.6358	Vibration of aromatic hydrocarbon C=C skeleton
10	1584.40	6.1295	10.5048	Vibration of aromatic hydrocarbon C=C skeleton
11	1623.93	6.6574	11.4093	Vibration of aromatic hydrocarbon C=C skeleton
12	1691.41	4.2350	7.2580	Conjugate C=O vibration
13	1749.07	1.4645	2.5099	Flexural vibration of carboxylic acid C=O

The peak fitting results of coal samples show that the FT-IR spectrum fitting of oxygen-containing functional groups in coal is complicated, with 13 fitting peaks. Among them, the absorption peak at 1098.95 cm^−1^ is C–O–C vibration, and the content is 7.13%. Four absorption peaks in the area from 1171.08 to 1223.10 cm^−1^ are attributed to the C–O vibration of phenol, alcohol, ether, and ester, accounting for 28.63% of the total area. The absorption peak at 1691.41 cm^−1^ belongs to the stretching vibration of conjugated C=O in coal, and the absorption peak at 1749.07 cm^−1^ is caused by the stretching vibration of carboxyl. The proportion of carbonyl and carboxyl groups in the total fitting area of the oxygen-containing functional groups spectrum is 7.25% and 2.51%, respectively. It can be seen that among oxygen-containing functional groups, phenol, alcohol, ether, and ester C–O groups are the main ones, the C–O–C and C=O groups are complementary, and the carboxyl content is relatively low.

#### Analysis of aliphatic hydrocarbon absorption zone

**Figure 6:**
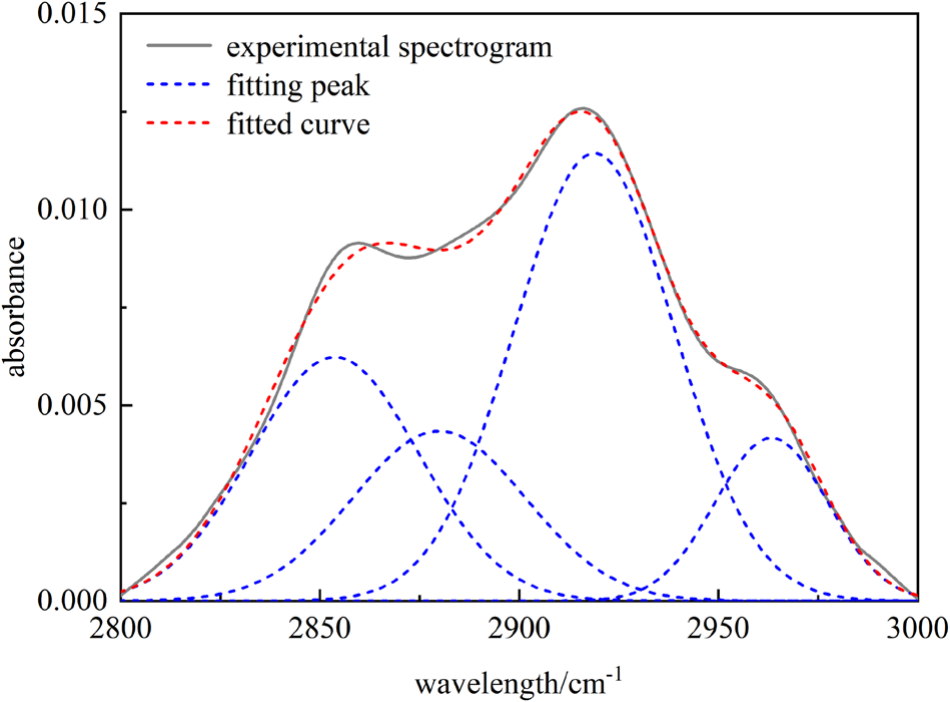
Fourier transform infrared spectroscopy fitting spectra of coal samples in the band 2800–3000 cm^−1^.

The wavelength range of the Fourier infrared spectrum of coal is 2800–3000 cm^−1^, which belongs to the absorption range of –CH_x_ in the aliphatic chains and alicyclic rings. Fit the spectrum of Chicheng coal sample in this band, as shown in Fig. 6, the area near peak 2950 cm^‒1^ belongs to asymmetric –CH_3_ stretching vibration, the area near peak 2920 cm^−1^ belongs to asymmetric –CH_2_ stretching vibration, and the area near peak 2850 cm^−1^ belongs to symmetric –CH_2_ stretching vibration, while the remaining functional groups belong to references^19,29^. The fitting parameters of the peaks are shown in Table 5.

**Table 5:** Infrared absorption peak parameters of aliphatic hydrocarbon from coal.

Peak number	Peak position (cm^−1^)	Peak area	Proportion (%)	Functional group
1	2853.72	1.1618	25.3672	Telescopic vibration of symmetric CH_2_
2	2879.89	0.8402	18.3461	CH telescopic vibration
3	2918.86	2.0555	44.8866	Telescopic vibration of asymmetric CH_2_
4	2963.30	0.5221	11.4001	Telescopic vibration of asymmetric CH_3_

The peak fitting results of the aliphatic hydrocarbon spectrum of coal samples show that four fitting peaks in the spectrum belong to four types of vibration absorption of three groups. Two main absorption peaks appear at 2853.72 cm^−1^ and 2918.86 cm^−1^, which belong to the telescopic vibration of symmetric –CH_2_ and asymmetric –CH_2_, respectively, accounting for 25.37% and 44.87%. Therefore, –CH_2_ is the most important one in the coal aliphatic hydrocarbons, and the total content of–CH and –CH_2_ in the studied coalis 88.59%. This indicates that there are more burning side chains in the coal. In constructing a coal macromolecular model, –CH_2_ should be the main one, and –CH_3_ and –CH should be the auxiliary ones.

#### Analysis of hydroxyl absorption band of coal samples

**Figure 7:**
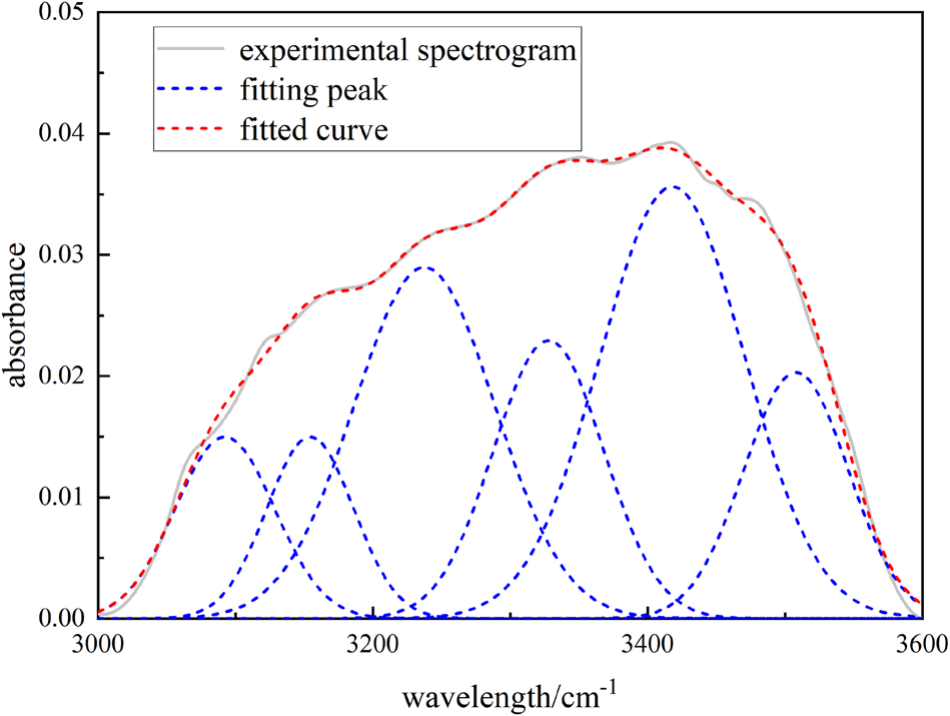
Fourier transform infrared spectroscopy fitting spectra of coal samples at bands 3000–3600 cm^−1^.

The hydroxyl group has an important influence on the reactivity of coal and is the main functional group to form the ammonia bond. The peak fitting results of coal samples in the band of 3000–3600 cm^−1^ are shown in Fig. 7 and Table 6. The structural types of hydroxyl groups mainly include OH‒N hydrogen bonds, cyclic hydrogen bonds, OH ether hydrogen bonds, OH‒OH hydrogen bonds, and OH‒π hydrogen bonds. The attribution of functional groups is determined according to references^19,29^.

**Table 6:** Absorption peak parameters of coal hydroxyl infrared spectrum.

Peak number	Peak position (cm^−1^)	Peak area	Proportion (%)	Functional group
1	3084.51	1.0518	6.92525	OH‒N hydrogen bond
2	3160.20	2.3460	15.4462	Cyclic hydrogen bonding
3	3247.40	2.6797	17.6438	Cyclic hydrogen bonding
4	3328.33	2.7724	18.2537	OH–O hydrogen bond
5	3410.37	3.5046	23.0748	OH–OH hydrogen bond
6	3499.34	2.8335	18.6562	OH–π hydrogen bond

The peak fitting results of the hydroxyl spectrum of coal samples show six peaks in the hydroxyl spectrum of coal and five types of hydroxyl groups. Among all the H bonds, cyclic hydrogen bonds and OH–OH hydrogen bonds are the main ones, with contents of 33.09% and 23.07%, respectively. They are supplemented by OH–O hydrogen bonds and OH–π hydrogen bonds, with contents of 18.25% and 18.65%, respectively. The OH‒N hydrogen bond content is the lowest, at 6.92%. The H bond contained in coal is an important symbol of the coal association model. The interaction force of the H bond is about one-tenth of that of the covalent bond, but it is about 10 times stronger than that of non-specific molecules. The existence of the H bond makes the coal macromolecular network more stable.

### X-ray photoelectron spectroscopy analysis

XPS, as an analytical method of organic molecular structure, can distinguish the existing state and relative content of carbon, oxygen, nitrogen, and sulfur in coal and is widely used in the research field of coal structure^30^.

**Figure 8:**
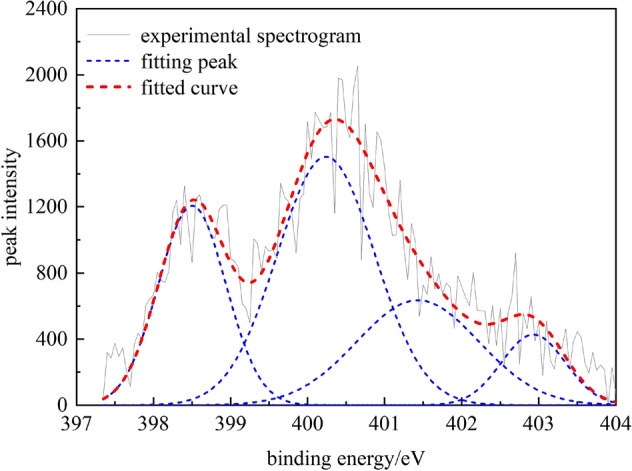
Peak fitting map of nitrogen in coal samples.

Analysis of nitrogen in coal
  When XPS is used to analyze the occurrence of nitrogen in coal, four characteristic peaks can be fitted, namely pyridine nitrogen (C_5_H_5_N) with a peak-position binding energy of 398.8 ± 0.4 eV, pyrrole nitrogen (C_4_H_5_N) with a peak-position binding energy of 400.2 ± 0.3 eV, quaternary nitrogen (-N-(CH_3_)_3_) with peak-position binding energy of 401.4 ± 0.3 eV and nitrogen oxide (N_x_O_y_). XPS data fluctuate greatly, so it is necessary to smooth the data before fitting the peaks. The fitting results are shown in Fig. 8 and Table 7.

**Table 7:** Form and content of nitrogen in coal samples.

Peak number	Peak position (cm^−1^)	Peak area	Proportion (%)	Functional group
1	398.50	1320.0326	24.4010	Pyridine nitrogen (C_5_H_5_N)
2	400.23	2388.7260	44.1559	Pyrrole nitrogen (C_4_H_5_N)
3	401.43	1259.0728	23.2741	Quaternary nitrogen(–N–(CH_3_)_3_)
4	402.93	441.9247	8.1690	Nitrogen oxide (N_x_O_y_)
  The peak fitting results of the XPS spectrum of nitrogen in coal samples suggest that the main forms of nitrogen in coal samples are pyrrole nitrogen (C_4_H_5_N) and pyridine nitrogen (C_5_H_5_N), the contents of which reach 44.16% and 24.4%, respectively; this is because both C_4_H_5_N and C_5_H_5_N belong to the aromatic conjugated system, which has high stability and can be preserved in the evolution of coal. This is followed by quaternary nitrogen (‒N‒(CH_3_)_3_) with a content of 23.27%, indicating a certain amount of pyridine nitrogen in the multiple aromatic structural units of coal molecules. The nitrogen oxides (NxOy) have a low content in coal samples (8.17%). They are mainly produced by the oxidation of C_5_H_5_N and C_4_H_5_N, so the relative content is relatively small.
(2) Analysis of sulfur in coal
  When XPS is used to analyze the occurrence state of sulfur in coal, the forms of sulfur can be divided into four categories: mercaptan, thioether, thiophene, sulfone, sulfoxide, and inorganic sulfur^24^. The corresponding electronic binding energy ranges are 162.2‒164 eV, 164‒164.4 eV, 165‒168 eV, and 169‒171 eV, respectively. In this paper, 162‒171 eV was selected as the analysis interval of the electron binding energy of sulfur, and the fitting results are shown in Fig. 9 and Table 8.

**Figure 9:**
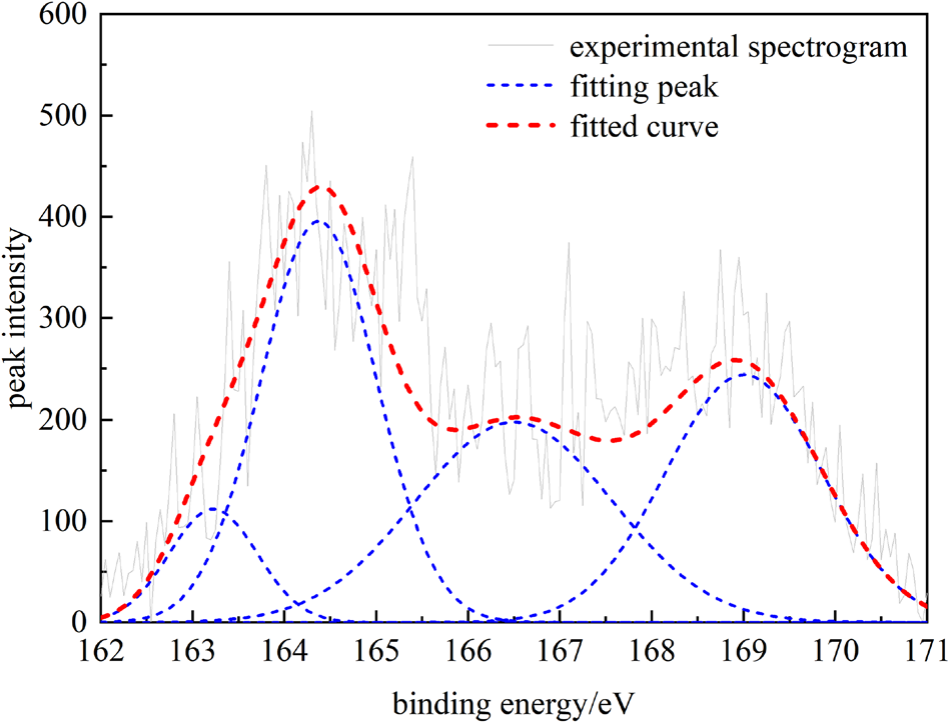
Sulfur peak fitting map of coal samples.

**Table 8:** Analysis results of sulfur elements in coal samples.

Peak number	Peak position (cm^−1^)	Peak area	Proportion (%)	Functional group
1	163.22	134.1273	7.4209	Thiols and thioethers
2	164.37	624.3158	34.5417	Thiophenes
3	166.50	531.1398	29.3866	Sulfone and sulfoxide
4	169.01	517.8419	28.6508	Inorganic sulfur
  The XPS spectrum peak fitting results of sulfur in coal samples suggest that thiophene is the main form of sulfur in coal, with a content of 34.54%, followed by sulfones, sulfoxides, and inorganic sulfur, with contents of 29.38% and 28.65%, respectively. Mercaptans and thioethershave a relatively low content of about 7.42%. This is because most functional groups of thioethers, thioesters, and thioethers are caused by the conversion or lossby the action of heat. At the same time, the degree of molecular fusion also increases and is transformed into a more stable structure. Thiophenes have the characteristics of aromatic structure and are one of the products of unstable side chain sulfur transformation. Therefore, thiophene has gradually become the main body of the organic sulfur structure.

### Nuclear magnetic resonance spectrum (^13^C NMR) analysis

The types of carbon atoms in the molecular structure of coal are mainly divided into aliphatic carbon and aromatic carbon. Different types of carbon atoms or functional groups connected to them and different connection methods lead to different chemical shifts of the corresponding peaks of ^13^C NMR spectra. ^13^C NMR spectrum provides quantitative and qualitative data about the macromolecular structure of coal and can be used to obtain coal carbon skeleton information directly and then study the macromolecular structure characteristics of coal^13,18^. According to the reference, the structural attribution of the chemical shift of carbon atoms in coal macromolecular structure is shown in Table 9.

**Table 9:** Attribution of carbon chemical shift^13^.

Chemical shift (ppm)	Functional group	Chemical shift (ppm)	Functional group
0‒25	Fatty (aryl) methyl/R(Ar)‒CH_3_/(*f*_al_*)	148‒165	Oxygen substituted aromatic carbon/Ar‒O/(*f*_a_^P^)
25‒50	Methylene, methylene, quaternary carbon	129‒165	Non-protonated aromatic carbon/(*f*_a_^N^)
	/CH_2_, CH, C/(*f*_al_^H^)	165‒190	Carboxyl carbon/COOH/(*f*_a_^C^)
50‒90	Oxygen-bonded fatty carbon/ O‒CH_3_(CH_2_)/(*f*_al_^O^)	190‒220	Carbonyl carbon/C=O/(*f*_a_^C^)
90‒129	Protonated aromatic carbon/Ar‒H/(*f*_a_^H^)	16‒90	Fatty carbon/(*f*_al_)
129‒137	Bridging aromatic carbon/(*f*_a_^B^)	90‒220	Total aromatic carbon/(*f*_a_)
137‒148	Side-branched aromatic carbon/Ar‒C/(*f*_a_^S^)	90‒165	Aromatic carbon ratio/(*f*_a_^’^)

**Figure 10:**
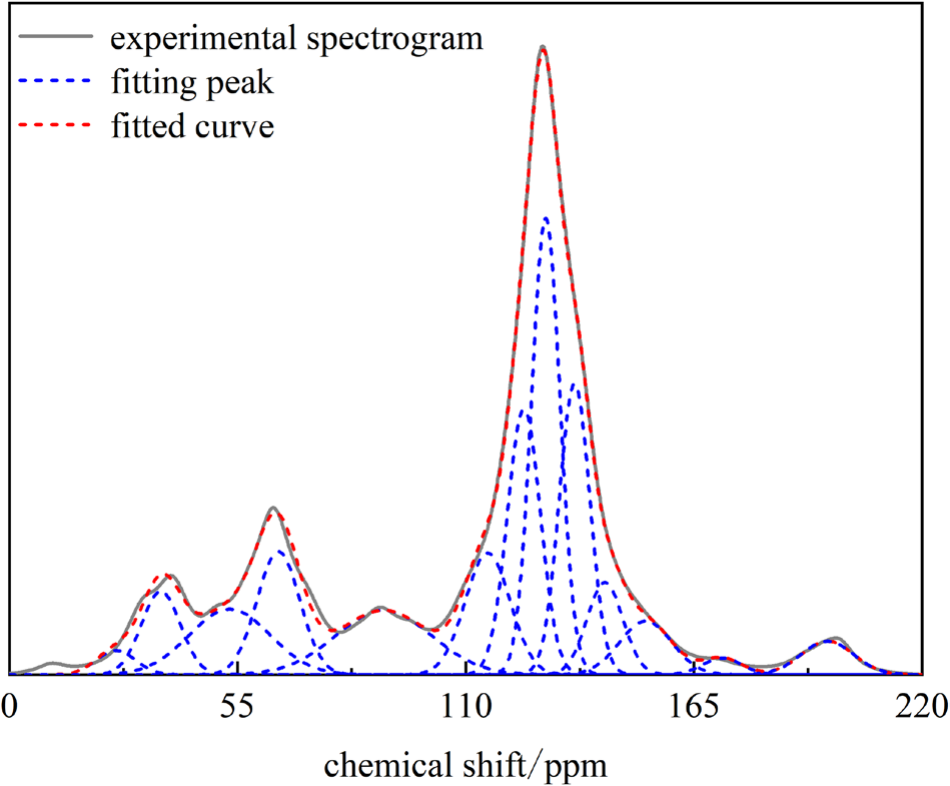
^13^C NMR spectra of coal samples.

Nuclear magnetic resonance studies the absorption of radio frequency energy by magnetic nuclei, which is suitable for the structural analysis of small molecules, large molecules, and complex systems. Because the structure of carbon functional groups in coal is highly complex, the peaks in the13C NMR spectrum overlap, so it is necessary to fit the spectrum to obtain the type and content of carbon functional groups corresponding to specific chemical shifts. In this paper, the ORIGIN software selected the chemical shifts of the 13C NMR spectrum of 0–200 ppm. Peak fitting on the test results from the nonsticky coal samples was performed, and the peak fitting results are shown in Fig. 10. According to the previous research results, the chemical shift values and assigned functional groups of different carbon structures in the 13C NMR spectra are summarized in Table 10.

**Table 10:** Parameters of ^13^C NMR peak splitting spectrum of coking coal.

Peak number	Chemical shift (ppm)	Proportion (%)	Functional group
1	24.71987	1.45606	Fatty (aromatic) methyl group
2	36.52321	4.93721	Methylene and quaternary carbon
3	53.14415	8.08438	Oxygen-bonded fatty carbon
4	64.86437	8.04621	Oxygen-bonded fatty carbon
5	92.24863	9.17054	Protonated aromatic carbon
6	115.29969	8.12066	Protonated aromatic carbon
7	124.02693	13.15583	Protonated aromatic carbon
8	127.28828	19.48695	Protonated aromatic carbon
9	135.23118	14.74507	Bridging aromatic carbon
10	143.55281	4.94291	Side-branched aromatic carbon
11	153.39552	4.1746	Oxygen substituted aromatic carbon
12	171.82287	0.99611	Carboxyl carbon
13	197.19926	2.68348	Carbonyl carbon

The fitting results indicate that the 13C NMR of coal samples mainly consists of two peak groups, 0–90 ppm and 90–165 ppm, namely fatty carbon (fal = fal* + falH + falO) and aromatic carbon (fa’ = faH + faN = faH + faP + faS + faB). The content of fatty carbon is about 22.52%, and the content of aromatic carbon is about 73.79%. The content of fatty carbon is mainly oxygen-bonded fatty carbon, accounting for about 16.12%, followed by methyl and quaternary carbon, accounting for about 4.94%/ The content of aromatic carbon is mainly protonated aromatic carbon, accounting for about 49.93%, supplemented by bridged aromatic carbon, accounting for about 17.74%. The overall contents of carboxyl carbon and carbonyl carbon in the coal samples are low, about 0.99% and 2.68%, respectively. The specific structural parameters are shown in Table 11.

**Table 11:** Structural parameters of ^13^C NMR of coal samples.

*f*_al_*	*f*_al_^H^	*f*_al_^O^	*f*_a_^H^	*f*_a_^B^	*f*_a_^S^	*f*_a_^P^	*f*_a_^N^	*f*_a_^C^	*f*_al_	*f*_a_	*f*_a_^’^	*f*_al_*
1.45	4.94	16.13	49.93	14.74	4.94	4.17	22.86	2.68	22.52	77.48	73.79	1.45

The ratio of bridging carbon to peripheral carbon (XBP) in the macromolecular structure of coal can characterize the polycondensation degree of aromatic structure in the structure^13^, and the calculation formula is as follows:

6 $$X_{BP} = \frac{{f_{a}^{B} }}{{f_{a}^{H} + f_{a}^{P} + f_{a}^{S} }}$$

Substituting the relevant parameters calculated in Table 10 into formula (6), the carbon ratio around the bridge of Chicheng Coal Mine was found as 0.25.

## Non-caking coal macromolecular model construction

The ratio of peribridge carbon of the benzene ring, pyridine, pyrrole, and thiophene sulfur in coal samples is 0; the peribridge carbon ratio of the naphthalene ring is 0.25; the peribridge carbon ratio of anthracene to phenanthrene is 0.4; the peribridge carbon ratio of pyrene and its isomers is 0.5^13,23,24^.

The ratio of bridge carbon to peripheral carbon of the coal sample is 0.25, so in this paper, benzene and naphthalene are the main aromatic carbon structures, and anthracene is the auxiliary. The types and quantities of aromatic structures closest to the bridge cycle ratio were obtained using Matlab calculations, as shown in Table 12. The total number of aromatic carbons in the model is 153. According to ^13^C NMR analysis, aromatic carbons account for 73.79%, so the total number of carbons in the coal molecular structure is 207, and then 54 aliphatic carbons and (carboxyl) carbonyl carbons can be obtained.

**Table 12:** The existence form of aromatic carbon in coal macromolecular configuration.

Existence form	Quantity	Existence form	Quantity
	Seven		One
	Eight		Two
	One		One

According to the proximate/ultimate analysis of coal samples, it is known that the carbon content of non-caking coal is 77.21%, the oxygen content is 15.66%, the nitrogen content is 1.32%, and the sulfur content is 1.05%. Accordingly, the amount of oxygen, nitrogen, and sulfur in the coal structure can be calculated as 32, 3, and 1, respectively. The XPS analysis allowed to determine that the main forms of N in long-flame coal are pyridine nitrogen and pyrrole nitrogen, and the quantity ratio is about 1:2, so there is one pyridine nitrogen and two pyrrole nitrogen. Sulfur is mainly thiophene sulfur, so coal molecules have a sulfur atom in the form of thiophene sulfur. According to FT-IR analysis, the ratio of C–O–C and phenol, alcohol, and ether (C–O) to carboxyl (COOH) and carbonyl (C=O) in oxygen-containing functional groups of coal is about 3.7:1, so the number of C–O–C and C–O groups is 25, and the number of carboxyl and carbonyl groups is 7. According to FT-IR analysis, the ratio of carboxyl to carbonyl groups is about 2.89:1. According to nuclear magnetic resonance analysis, the ratio of carboxyl to carbonyl groups is about 2.71:1. Therefore, the number of carboxyl groups in the coal sample is 1, and the number of carbonyl groups is 5. Combined with the 13C NMR test, the ratio of oxygen substitution to oxygen grease content is about 3.8:1.

**Figure 11:**
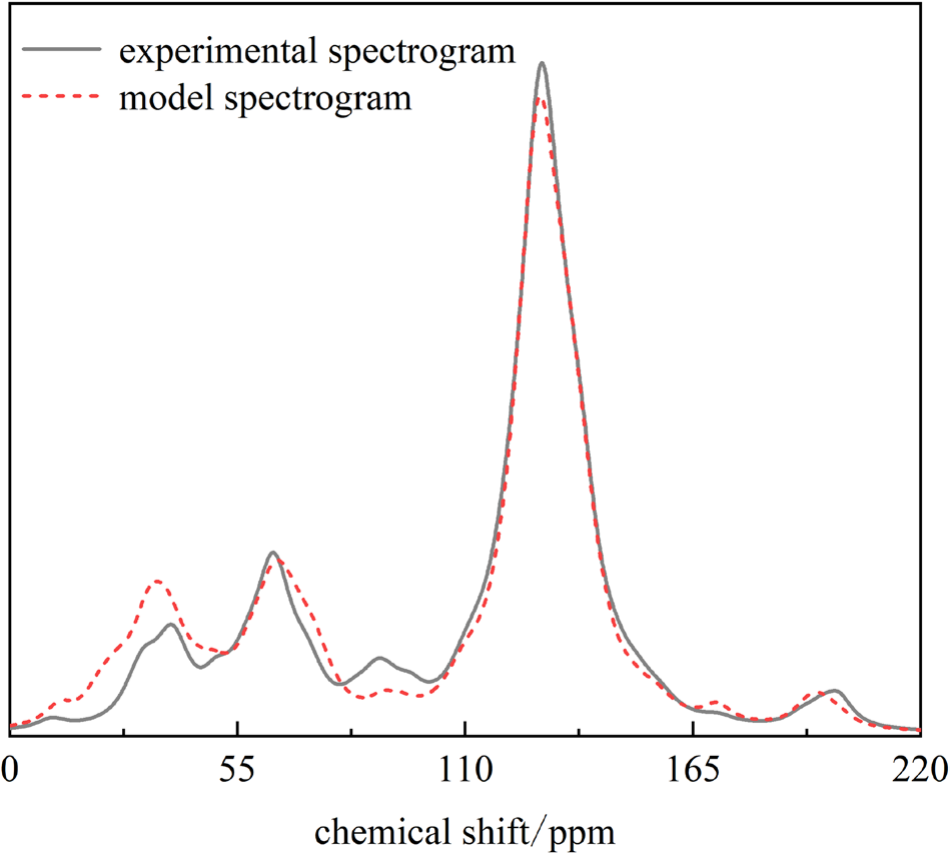
Comparison of experimental ^13^C NMR spectra and model calculated spectra of coal.

**Figure 12:**
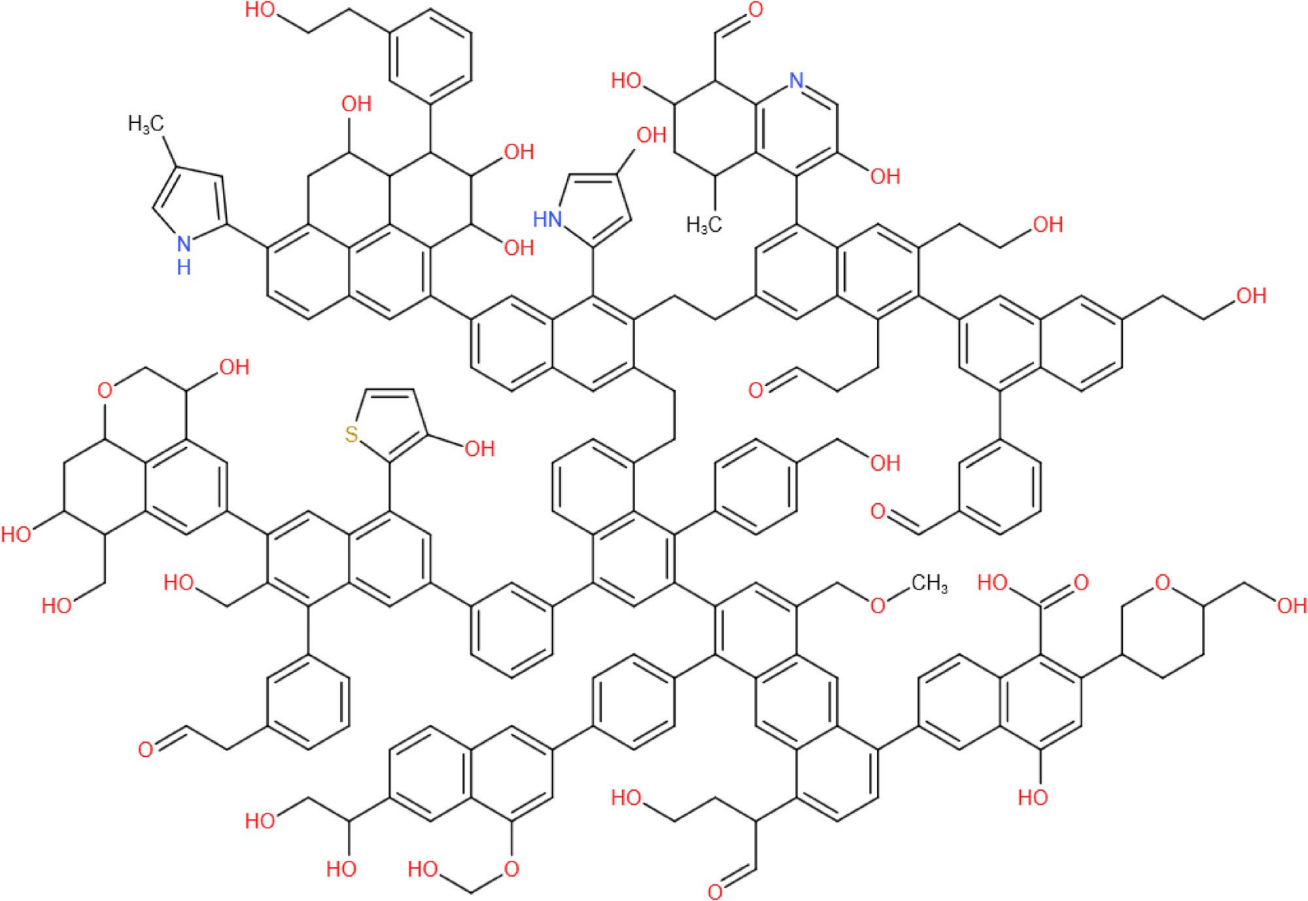
Plane model of coal macromolecular structure.

According to the above results, a non-caking coal macromolecular model was established by using Kingdraw chemical drawing software, and then the non-caking coal macromolecular model was imported into MestReNova software. By adjusting the positions and connection modes of different functional groups in the coal macromolecular model, the ^13^C NMR spectrum of the model was finally compared with the experimental ^13^C NMR spectrum, as shown in Fig. 11. The results show that the ^13^C NMR spectrum of the established coal macromolecular model is in good agreement with the experimental test spectrum. Finally, it is determined that the molecular formula of the non-caking coal in Chicheng Coal Mine is C_207_H_181_O_32_N_3_S(C: 76.39%, N: 1.29%, O: 15.73%, H: 5.61%, S: 0.99, which is close to the test results of proximate/ultimate analysis of experimental coal samples), and the coal macromolecular model is shown in Fig. 12.

The functional groups in coal have an impact on the natural propensity of coal, its gas adsorption characteristics, as well as its combustion, cracking, and coking performance. When the content of oxygen-containing functional groups in coal is high, the chemical properties of hydroxyl, carboxyl, and ether bonds are active and prone to oxidation reaction with oxygen. Coal is more inclined to low-temperature oxidation, with a higher natural propensity. Moreover, the aromatic hydrocarbon structure in coal is stronger than that of ether bonds, and the carboxyl group has a stronger adsorption ability for methane molecules, This is the reason why higher rank coal adsorbs more methane. The coking and combustion performance of coal are different, and the coal rank has different reactions, which are essentially different from coal.

## Conclusions

In this paper, through proximate/ultimate analysis, FT-IR, XPS, and ^13^C NMR characterization experiments, the distribution of oxygen-containing functional groups, fat structure, and aromatic structure of coal was obtained, and the macromolecular structure model of low-rank coal was constructed. The main conclusions were as follows. The obtained model provides a model basis for further research on the spontaneous combustion tendency and gas adsorption capacity of Chicheng non caking coal, better understanding of Chicheng coal, and providing theoretical support for subsequent clean and efficient utilization.

1. Through proximate/ultimate analysis and FT-IR structure analysis and calculation, the elemental content of the Chicheng coal mine coal sample was obtained. The fatty chain length of the coal sample was 0.17, the aromaticity was 0.83, the aromatic ring condensation degree was 0.18, and the infrared aromatic carbon rate was 0.7764.
2. Using XPS analysis, it was concluded that the main forms of nitrogen in the coal samples were pyrrole nitrogen (C_4_H_5_N) and pyridine nitrogen (C_5_H_5_N), with corresponding contents of 44.16% and 24.4%. The main forms of sulfur were thiophene, with a content of 34.54%, supplemented by sulfones, sulfoxides, and inorganic sulfur, with contents of 29.38% and 28.65%, respectively. The content of mercaptans and thioethers was relatively low, about 7.42%.The aromatic carbon rate of nuclear magnetic resonance was 0.7379, close to the aromatic carbon rate measured by the infrared spectrum, and the ratio of bridge carbon to peripheral carbon of the coal sample was 0.25.
3. Based on the proximate/ultimate analysis, FT-IR, XPS, and ^13^C NMR experiment results, a molecular plane model of non-caking coal in Chicheng Coal Mine was established, which was in good agreement with the experimental ^13^C NMR spectrum. The molecular structural formula of coal was C_207_H_181_O_32_N_3_S, which was close to the test results of proximate/ultimate analysis of experimental coal samples, indicating the rationality of the model.
4. Compared to high-order anthracite, the Chicheng non-caking coal constructed in this article contains more oxygen-containing functional groups, with the highest hydroxyl content. The aromatic hydrocarbon structure is mainly benzene and naphthalene, while the aromatic structure of high-order anthracite is mainly anthracene and phenanthrene. Compared to high-order anthracite, Chicheng non-caking coal contains a large amount of alicyclic and aliphatic chain structures, exhibiting typical low order coal macromolecular structural characteristics.
